# MicroRNA-135a regulates NHE9 to inhibit proliferation and migration of glioblastoma cells

**DOI:** 10.1186/s12964-017-0209-7

**Published:** 2017-12-21

**Authors:** Daniela M. Gomez Zubieta, Mohamed A. Hamood, Rami Beydoun, Ashley E. Pall, Kalyan C. Kondapalli

**Affiliations:** Department of Natural Sciences, University of Michigan-Dearborn, 4901 Evergreen Road, SFC # 207, Dearborn, MI 48128 USA

**Keywords:** Sodium-proton exchange, NHE9, SLC9A9, pH, Endosome, Glioma, microRNA, Cancer, Neurological disease, Glioblastoma, Epidermal growth factor receptor (EGFR), miR-135a, miR-124

## Abstract

**Background:**

Glioblastoma multiformae (GBM) is the most aggressive type of malignant brain tumor with complex molecular profile. Overexpression of Na^+^/H^+^ Exchanger isoform 9 (NHE9) promotes tumor progression and correlates positively with insensitivity to radiochemotherapy and poor prognosis. However, molecular mechanisms responsible for increase in NHE9 levels beyond a critical threshold have not been identified.

**Methods:**

Bioinformatics analysis, luciferase reporter assays, real-time PCR and western blotting were conducted to examine the expression profiles and identify microRNAs (miRNA) that target NHE9. Cell proliferation and migration assays were conducted in U87 glioblastoma cells to determine the consequence of miRNA mediated targeting of NHE9. Endosomal pH measurements, immunofluorescence microscopy and surface biotinylation experiments were conducted to characterize the mechanistic basis of regulation.

**Results:**

We show that microRNA 135a (miR-135a) targets NHE9 to downregulate its expression in U87 cells. MiR-135a levels are significantly lower in glioblastoma cells compared to normal brain tissue. Downregulation of NHE9 expression by miR-135a affects proliferative and migratory capacity of U87 cells. Selectively increasing NHE9 expression in these cells restored their ability to proliferate and migrate. We demonstrate that miR-135a takes a two-pronged approach affecting epidermal growth factor receptors (EGFRs) to suppress tumor cell growth and migration. EGFR activity is a potent stimulator of oncogenic signaling. While miR-135a targets EGFR transcripts to decrease the total number of receptors made, by targeting NHE9 it routes the few EGFRs made away from the plasma membrane to dampen oncogenic signaling. NHE9 is localized to sorting endosomes in glioblastoma cells where it alkalinizes the endosome lumen by leaking protons. Downregulation of NHE9 expression by miR-135a acidifies sorting endosomes limiting EGFR trafficking to the glioblastoma cell membrane.

**Conclusions:**

We propose downregulation of miR-135a as a potential mechanism underlying the high NHE9 expression observed in subset of glioblastomas. Future studies should explore miR-135a as a potential therapeutic for glioblastomas with NHE9 overexpression.

**Electronic supplementary material:**

The online version of this article (10.1186/s12964-017-0209-7) contains supplementary material, which is available to authorized users.

## Background

Glioblastoma multiformae (GBM) are the most lethal primary brain tumors [1, 2]. Characterized by rapidly invasive growth pattern and high angiogenesis, complete surgical resection of GBM is extremely difficult [3]. Even with surgery and radiochemotherapy the median survival for patients is 12–15 months [3]. Accumulating evidence suggests molecular heterogeneity is a key hurdle in improving clinical outcomes [4–6]. Effective treatment strategies should therefore be tailored to a patient’s unique tumor genetic makeup [4, 5]. Identifying specific genetic subsets of tumors and using these as therapeutic targets is now a major focus of glioma research [7]. In GBM, increase in Na^+^/H^+^ Exchanger NHE9 protein levels has recently been identified as a potent driver of tumor progression and is associated with decreased patient survival [7].

SLC9A9 gene encoding NHE9 is one of the top 12% of the genes overexpressed in GBM [7]. Following neo-adjuvant chemotherapy and tumor resection, GBM patients overexpressing NHE9 remained disease free for ~11 months with median survival of only 16 months compared to patients with unaltered expression of NHE9 who remained disease free for ~34 months with median survival of ~59 months [7]. Moreover, overexpression of NHE9 in GBM correlates positively with radiochemotherapy insensitivity [7, 8]. In glia, NHE9 is localized to sorting endosomes [9] where it regulates endosomal pH by transporting protons out in exchange for sodium or potassium ions [10–13]. Overexpression of NHE9 alkalinizes the sorting endosomes and thereby affects sorting of epidermal growth factor receptors (EGFRs) [7]. EGFR signaling regulates multiple cellular functions including cell growth and division [14]. Dysregulated EGFR activity is characteristic of GBM oncogenic signature [15]. Endocytosis of EGFRs removes the receptors from the membrane following which they are routed to the lysosome, thereby terminating the signaling [16]. Increased NHE9 activity redirects EGFR to the plasma membrane resulting in activation of downstream oncogenic signaling pathways [7]. The mechanisms underlying overexpression of NHE9 in GBM have not been studied. In wake of the emerging roles in cancer, we focused on the microRNA (miRNA) mediated regulation of NHE9 and EGFR-dependent oncogenic signaling.

MicroRNAs are 19–24 nucleotide single-stranded noncoding RNAs that can regulate gene expression at both post-transcriptional and translational levels [17]. MiRNAs interact specifically with 3’untranslated regions (UTRs) of messenger RNA (mRNA) to decrease the stability of mRNAs leading to reduced expression of protein [17]. Deregulation of miRNA expression has been observed in many cancers including glioma [18–20]. In comparison to normal brain, deregulation of more than 290 miRNAs has been reported in GBM [18]. It is well established in preclinical models that restoring miRNA levels can inhibit tumor growth, consistent with functional role for miRNAs as tumor suppressors [21]. However, each miRNA is capable of targeting multiple genes [22–24]. Manipulation of miRNA levels can have implications on biological processes globally. Therefore, miRNAs that are enriched in or specific to tissue of interest are potentially promising candidates for therapy. Several therapeutic miRNA mimics are in preclinical and clinical stages of development [21, 25, 26]. MiRNAs are stable in tissue and body fluids and could also serve as non -invasive biomarkers of cancer [27]. Identifying miRNAs that target NHE9 transcript in glia thus hold great promise for a subset of GBM patients.

In this study, we identified miR-135a as a regulator of NHE9 protein expression. MiR-135a, a glial cell enriched miRNA, is known to be downregulated in glioma [28]. Here, we examined the regulation of NHE9 by miR-135a in a well-characterized GBM cell model. We demonstrate that miR-135a downregulates EGFR and NHE9 protein expression to attenuate membrane turnover of EGFRs to impair GBM cell growth and ability to migrate. We propose a potential therapeutic role for miR-135a in GBM with NHE9 overexpression.

## Methods

### Glioma cell lines, plasmids and miRNA mimics

U87 cells were obtained from Dr. Alfredo Quiñones-Hinojosa’s laboratory (Johns Hopkins University, currently at Mayo Clinic). U251n cells were obtained from Dr. Feng Jiang’s laboratory (Henry Ford Hospital). U87 and U251 cells were maintained in DMEM media (Invitrogen) supplemented with 10% fetal bovine serum (Sigma), and 5% Antibiotic-Antimycotic (10,000 U/ml penicillin, 10,000 mg/ml streptomycin, Gibco). Cells were maintained in a 5% CO2 incubator at 37 °C. The growth medium was completely exchanged with fresh medium twice a week. Micro-RNA mimics, hsa-miR-135a-5p, hsa-153-3p and hsa-124-3p as well as the scrambled control (miRIDIAN microRNA Mimic Negative Control #1) were purchased from Dharmacon. Transfection effeciencies were optimized using miRIDIAN microRNA Mimic Transfection Control with Dy547 **(**Dharmacon**).** Full-length mNHE9-EGFP and mNHE9-mcherry were cloned into FuGW lentiviral vector as previously described [9]. Empty vector (FuGW) was used for control transductions. Viral Core Facility of the University of Michigan executed lentiviral packaging of the virus.

### Computational analysis

MiRNAs targeting NHE9 transcript were predicted through the algorithms TargetScan Human 5.2 (http://www.targetscan.org) [29]. We obtained miRNA abundance in various human tissues from the human miRNA tissue atlas (https://ccb-web.cs.uni-saarland.de/tissueatlas/) [30]. SLC9A9 transcript abundance in various tissues was obtained from published RNA-seq analysis of tissue samples from 95 human individuals (https://www.ncbi.nlm.nih.gov/gene/285195/?report=expression) [31]. Comparative analysis for miR-135a and NHE9 mRNA expression in various tissues is presented as percent relative to tissue of highest expression.

### Luciferase reporter assay

One thousand four hundred ninety three base pairs of SLC9A9 (NM_173653) 3’UTR cloned in pMIR target vector, with firefly luciferase as reporter, was purchased from Origene (SC214935). Luciferase assays were carried out in HEK 293 and U87 cells. Cells were co-transfected with mimics hsa-miR-135a-5p, hsa-153-3p and hsa-124-3p or scrambled control mimic in 24-well plates. The cells were then harvested and lysed for luciferase assay 48 h after transfection. Luciferase assays were performed using a luciferase assay kit (Promega) according to the manufacturer’s instructions.

### RNA isolation and qPCR

Total RNA from human brain tissue was obtained from Agilent Technologies. MiRNA was extracted from U87 and U251n cells using miRNeasy kit (Qiagen) following manufacturer’s instructions. cDNA was synthesized using miScript II RT kit (Qiagen) according to the manufacturer’s instructions. Quantitative real-time PCR analysis experiments were set up using miScript SYBR Green PCR Kit (Qiagen) according to manufacturer’s instructions on CFX connect real time system (Bio-Rad Laboratories). MiScript Primer Assay probes used were: MS00008624 (has-miR-135a-5p) and MS00033705 (Hs_SNORD61_11). mRNA was isolated from U87 cells using the RNeasy Mini kit (Qiagen) following manufacturer’s instructions as previously described. Taqman gene expression assay probes used were: Hs02758991_g1 and Mm99999915_g1 (GAPDH), Hs00543518_m1 and Mm00626012_m (NHE9). Cycle threshold (C_t_) values were first normalized to endogenous controls. Fold change was calculated as 2^−ΔΔCt^, where ΔΔCt is the normalized cycle threshold value relative to control. At least three technical replicates of three biological replicates were run to account for variance in assays.

### Indirect immunofluorescence

U87 cells on coverslips were washed twice with phosphate buffered saline (PBS). The cells were then fixed for 30 min at room temperature with solution containing 4% PFA and 4% sucrose in PBS, following previously published protocol [32]. For EGFR sorting experiments, cells were starved and then stimulated with 20 ng mL^−1^ EGF for 1 h at 37 °C before fixing. Fixing solution was removed by washing with PBS. Next, the cells were incubated for a half-hour in block solution (1%BSA, 0.3 M glycine, and 0.1% tween 20). For co-localization experiments with NHE9-GFP, Rab 5 antibody (Cell Signaling Technology) was diluted 1:100 in block solution without tween 20 and incubated overnight at 4˙C. Following PBS washes, Alexa Fluor conjugated secondary antibody (Invitrogen, USA) was used at 1:1000 dilutions for 30 min. Cells were mounted onto slides using Prolong gold antifade reagent (Invitrogen) and were imaged using Lumascope-620 microscope (Etaluma).

### qPCR analysis

mRNA was isolated using the RNeasy Mini kit (Qiagen) following manufacturer’s instructions with an additional step to remove DNA using DNase I (Ambion, Thermo fisher Scientific). cDNA was synthesized using the High-Capacity RNA-to-cDNA Kit (Applied Biosystems) following manufacturer’s instructions. Quantitative real-time PCR analysis experiments were set up using Taqman fast universal PCR Master Mix (Applied Biosystems) according to manufacturer’s instructions on CFX connect real time system (Bio-Rad Laboratories). Taqman gene expression assay probes used were: Hs02758991_g1 and Mm99999915_g1 (GAPDH), Hs00543518_m1 and Mm00626012_m (NHE9), 1Hs00951083_m1 (TfR), Hs03003631_g1 (18 s rRNA). Cycle threshold (C_t_) values were first normalized to endogenous controls. Fold change was calculated as 2^−ΔΔCt^, where ΔΔCt is the normalized cycle threshold value relative to control. Three technical replicates of three biological replicates were run to account for variance in assays.

### Surface Biotinylation and western blotting

U87 cells were EGF starved for 2 h. Following which, 20 ng mL^−1^ EGF was added to the media for 30 min and surface proteins were labeled with biotin as previously described [9, 32]. Briefly, cells were washed with ice-cold PBS at least 3 times and incubated with Sulfo-NHS-LC-biotin at 1 m/ml in PBS for 20 min at 4 °C. Excess NHS groups were quenched using glycine (100 mM). Cells were lysed with Mammalian Protein Extraction Reagent (M-PER, Thermo Fisher Scientific) that included protease inhibitor cocktail (Halt Protease Inhibitor Cocktail, Thermo Fisher Scientific) and separated by SDS-polyacrylamide gel electrophoresis as previously described. NHE9 antibody used for western blotting was purchased from ProteinTech (catalog #13718–1-AP) and EGFR antibody was purchased from Millipore (catalog# 06–847). Both these antibodies were used at 1:100 dilutions. Loading control used was tubulin (Sigma T 9026, 1:1000).

### Cell proliferation assay

Cell proliferation was quantified using MTS assay (Celltiter 96 Aqueous One Solution Cell Proliferation Assay, Promega). This is a calorimetric assay, which works on the principle of Owens reagent being converted into a colored formazan product by viable cells. Briefly, 1 × 10^4^ cells were seeded, in triplicate, into a 96-well plate. U87 cells plated in 96 well plates were transfected with 100 nm of miR-135a mimic or the control using Lipofectamine LTX (Thermo Fisher Scientific). For NHE9 overexpression, U87 cells were transduced with lentiviral vector expressing NHE9-GFP as described previously [32]. MiRNA mimics were transfected in these cells 24 h after transduction. At each time point, 20 μl of Celltiter 96 Aqueous One Solution reagent was added to each well and incubated for 1 h at 37 °C. The absorbance was measured at 490 nM using a microplate reader (BioRad, iMark) and normalized to the samples absorbance on day 1.

### Cell migration assay

Oris Migration Assay (Platypus Technologies) was used to monitor U87 cell migration. Briefly, U87 cells were seeded in a 96 well plate (3.75 × 10^5^ cells per well) with a physical barrier (Silicone stopper) to create a central cell-free detection zone in the center of each well. 48 h later cells were then transfected with 100 nM miR-135a or control mimic using Lipofectamine LTX (Thermo Fisher Scientific). After 24-h, the stopper was removed and cells were incubated with live cell nuclear counterstain NucBlue (Thermo Fisher Scientific) for 20 min before monitoring the migration process. Fluorescence images were taken with Lumascope 620 (Etaluma) at various time points as indicated. For NHE9 overexpression, U87 cells were transduced with FUGW vector expressing NHE9-GFP as described previously. miRNA mimics were transfected in these cells 24 h after transduction.

### Endosomal pH measurement

U87 cells plated in fluorodishes (World Precision Instruments, FL, U.S.A.) were transfected with 100 nM miR-135a or control mimic. 48 h after transfection the fluorodishes were placed on ice for 10 min and then rinsed with cold imaging buffer (Live Cell Imaging Solution (Thermo Fisher Scientific) with 20 mM Glucose and 1% BSA) to remove residual transferrin. Cells were then incubated with 50 μg/ml fluorescein conjugated transferrin (Tfn-FITC, Thermo Fisher Scientific) in imaging buffer for 10 min. The cells were then rinsed with LCIS and fluorescence images were acquired (excitation 494 nm and emission 518 nm) with Lumascope 620 (Etaluma). Internal fluorescence was quantified using ImageJ [33] software, and average fluorescence intensity was recorded. To normalize for total transferrin uptake, pH insensitive 50μg/ml Alexa Fluor 568-conjugated transferrin (Tfn-568) was loaded as described above in both control and mir-135a transfected cells. Endosomal pH was determined from a standard curve that was generated using pH calibration buffer kit (Thermo Fisher Scientific). Briefly, cells were incubated with 50 μg/ml fluorescein conjugated transferrin for 10 min as described above and rinsed with imaging buffer. The cells were then loaded with 10 μM Cell Loading Solution (Thermo Fisher Scientific) that included Valinomycin and Nigericin (10 μM each) and incubated with calibration buffers of varying pH for 5 min at 37˙C, before fluorescence imaging.

## Results

### MiR-135a targets NHE9 and downregulates its expression

**Fig. 1:**
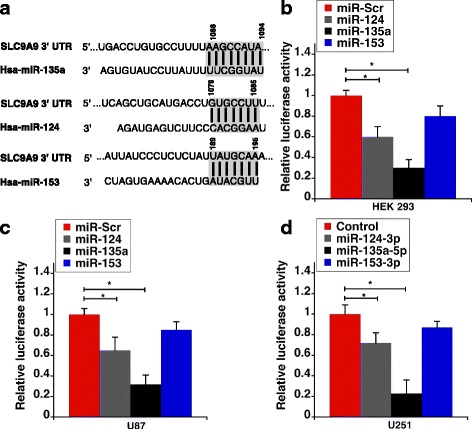
SLC9A9 transcript is a target of miR-135a-5p and miR-124-3p. **a** Seed sequences of miR-135a-5p, miR-124-3p and miR-153-3p along with their predicted binding sites in SLC9A9 3’-UTR are shown. The highlighted regions represent the binding sites as predicted by TargetScan. **b** MiR-135a-5p and miR-124-3p target the predicted binding sites within the transcript of SLC9A9 3’-UTR, decreasing luciferase activity in HEK293 cells transfected with a luciferase reporter. Relative luciferase activity normalized to protein concentration is plotted. **c** Luciferase activity as described in (**b**) is shown for U87 cells (**d**) Luciferase activity as described in (**b**) is shown for U251n cells. Error bars represent standard deviation (SD) determined from at least three biological replicates; *p < 0.05. Statistical analysis was done using student’s t-test

The most important feature for target recognition by microRNAs (miRNAs) is a 6–8 neucleotide long sequence on the miRNA known as the seed sequence [34]. Seed sequences in miRNAs are known to interact with target gene at multiple sites, both canonical and non-canonical [35]. However, it has been shown that pluralities of miRNA-mRNA interactions that result in translational repression occur via canonical and conserved sites [36]. As a first step to identify miRNAs that target NHE9 we used an online miRNA target prediction program TargetScan [29]. This program considers mRNA binding site type and conservation among other features to predict targets. TargetScan predicted miR-135a-5p, 153-3p and 124-3p as the most promising candidates. These three miRNAs are broadly conserved among vertebrates and have either a seven (miR-124-3p, 153-3p) or eight (miR-135a-5p) nucleotide complementarity between miRNA seed region and the 3’UTR of NHE9 transcript (Fig. 1A). Though length of seed region alone does not influence mRNA repression, prediction specificity increases with seed length [37].

**Fig. 2:**
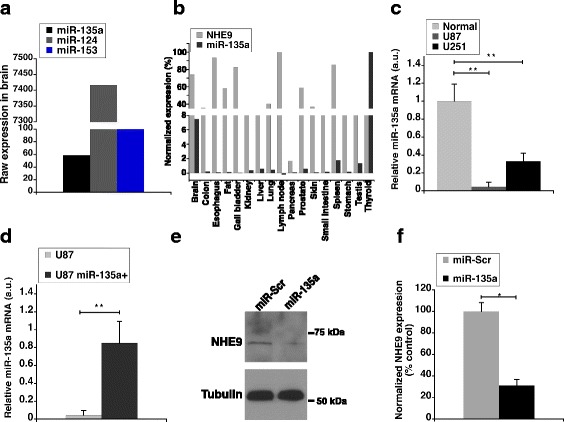
miR-135a-5p downregulates NHE9 protein expression. **a** Raw expression values of miR-135a, miR-124 and miR-153 were obtained from human miRNA tissue atlas. MiRNA abundance in brain from tissue biopsies of two normal (i.e. cancer free) individuals was conducted using SurePrint 8 × 60 K Human V19 and V21 miRNA microarray analysis as previously described [30]. **b** Comparative analysis of NHE9 mRNA and miR-135a expression profiles across various tissues. NHE9 mRNA expression profile was obtained from RNA-seq analysis performed of human tissue samples from 95 normal (i.e. cancer free) individuals as described previously [31]. MiRNA expression profile was obtained from human miRNA tissue atlas [30]. Normalization was done as a percentage relative to tissue with highest expression. **c** Mir-135a expression levels in U87 and U251n cell lines relative to normal human brain tissue. **d** qPCR analysis of miR-135a from U87 cells transfected with miR-135a mimic relative to control U87 cells. **e** Immunoblots of U87 cell lysates transfected with miR-135a or scrambled control mimic were probed using anti-NHE9 and anti-tubulin antibodies. **f** NHE9 protein expression levels determined by western blotting. Graphs represent average band intensity from densitometric scans of immunoblots from three biological replicates. Error bars represent standard deviation (SD), *p < 0.05 and **p < 0.01. Statistical analysis was done using student’s t-test

Next, we sought to experimentally validate interactions of the three miRNAs with the 3’UTR of NHE9. For this, a vector with the entire 3’UTR region of NHE9 connected at the downstream of a luciferase reporter gene was co-transfected with miR-124, 135a, 153 mimics or scrambled miRNA control in HEK293T cells. As shown in Fig. 1B, miR-135a and miR-124 suppressed luciferase activity by ~ 70% and 40% respectively relative to the scrambled control. Though we did see a ~25% reduction in luciferase activity with miR-153, under our test conditions, the variation failed to achieve statistical significance. A similar trend was observed when the assay was repeated in U87 (Fig. 1C) and U251n glioblastoma cells (Fig. 1D **)**. These results indicate that NHE9 is a direct target of miR-135a and miR-124. MiRNA abundance data of normal adult human brain obtained from the miRNA tissue atlas indicates that miR-124 levels are ~125 and ~60 fold higher than miR-135a and miR-153 respectively (Fig. 2A) [30]. Interestingly, miR-135a is enriched specifically in glial cells and is dramatically downregulated in gliomas [28]. Previous studies in prostate cancer cells also suggest a role for miR-135a in downregulation of epidermal growth factor mediated oncogenic signaling [38]. Therefore, we decided to focus on miR-135a in this study. Analysis of published expression profiles of miRNA-135a and NHE9 mRNA from various human tissues, as shown in Fig. 2B, revealed a strong negative correlation consistent with mRNA degradation mechanism involved in miR-135a–NHE9 interactions.

To investigate the role of miR-135a in glioblastoma progression driven by NHE9, we first compared the expression levels of miR-135a in glioblstoma cell lines (U87 and U251n) with total RNA from normal human brain (frontal cortex). MiR-135a expression levels were significantly lower in tumor cell lines compared to total RNA from normal brain tissues (Fig. 2C). Expression of miR-135a in U87 and U251 glioblastoma cell lines were 4.5% and 33% of normal brain tissue, respectively. This data supports the idea of a tumor suppressor role for miR-135a in glioblastoma. In the context of NHE9 protein expression driving oncogenic signaling in glioma, we sought to test whether miR-135a downregulates NHE9 protein levels in its role as a tumor suppressor. Transfection of U87 cells with miR-135a mimic increased miR-135a by 20 -fold in U87 cells (Fig. 2D). Western blotting at 72 h after transfection with miR-135a or scrambled control indicated NHE9 protein levels were reduced by ~3.2 fold when transfected with miR-135a compared to miRNA control in U87 cells (Fig. 2E and F). These data clearly indicate that miR-135a targets NHE9 to downregulate its expression.

### MiR-135a inhibits glioblastoma cell proliferation and migration by modulating NHE9 levels

**Fig. 3:**
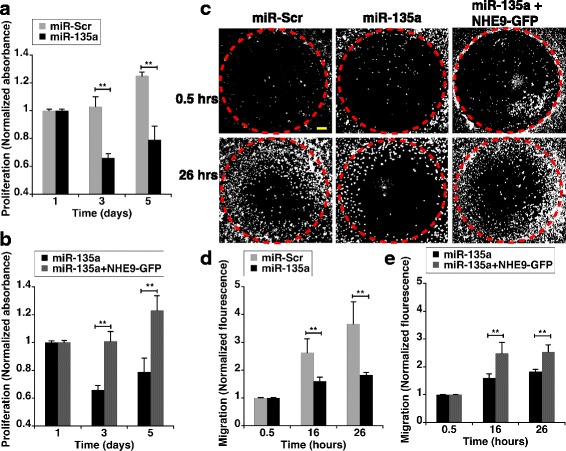
Downregulation of NHE9 expression via miR-135a affects U87 cell proliferation and migration. **a** Proliferation of U87 cells transfected with miR-Scr or miR-135a were determined by MTS assay. Absorbance at 490 nm for each sample was normalized to the samples absorbance on day 1. **b** Loss of proliferative ability in miR-135a transfected U87 glioma cells is reversed by ectopic expression of NHE9-GFP. Proliferation determination and normalization were done as described in (**a**). **c** Representative images of U87 cell migration assay taken at 0.5,16 and 26 h. Migration of U87 cells into cell free zones (red dashed-circles) was detected using live cell nuclear counterstain NucBlue (white dots). Loss of migration capacity in miR-135a transfected U87 glioma cells is reversed by ectopic expression of NHE9-GFP. Scale bar (yellow line) is 250 μm. (**d** and **e**) Graphs represent normalized fluorescence intensity from NucBlue staining of live cells migrating into the cell free zones at various time points as indicated. Fluorescence intensity of each sample was normalized to the samples fluorescence at 0.5 h. Error bars represent standard deviation (SD); **p < 0.01. Statistical analysis was done using student’s t-test. Graph represents an average of at least three biological replicates

MiR-135a has been reported to suppress the proliferation of glioma cells [28]. Consistent with previous observations, evaluation of proliferation capacity by MTS assay demonstrated that transfection of miR-135a reduced proliferation of U87 cells by ~34% relative to control in 48 h and ~40% in 96 h, after transfection (Fig. 3A). However, the proliferative ability was regained by co-expression with NHE9-GFP (Fig. 3B). This indicates NHE9 plays a role in miR-135a mediated inhibition of glioblastoma cell growth. NHE9 expression in U87 cells significantly increases migratory potential of U87 cells (Additional file 1: Figure S1). MiR-135a inhibited migration of U87 glioblastoma cells by ~50% relative to the control group, in 26 h (Fig. 3C left and middle panels and 3D). Similar to cell proliferation, ability to migrate was restored in cells when NHE9 protein levels were increased (Fig. 3C middle and right panels and 3E). Together, these data indicate that miR-135a regulates proliferation and migration in U87 glioblastoma cells via NHE9.

### Downregulation of NHE9 expression via miR-135a alters pH of sorting endosomes to affect EGFR trafficking in glioblastoma cells

**Fig. 4:**
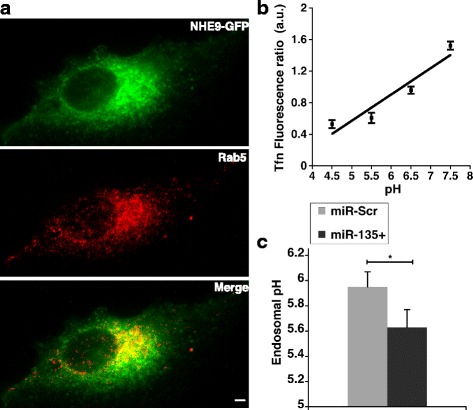
miR135a acidifies sorting endosomes by downregulating NHE9. **a** NHE9-GFP colocalizes with Rab5 in sorting endosomes of U87 cells as determined by immunofluorescence microscopy. *Top panel*: NHE9-GFP (*green*) *Middle panel:* sorting endosome marker, Rab5 (*red*) and *Bottom panel*: Merge. Colocalization is indicated by *yellow* in the *merge.* Scale bar is 10 μm. Quantification of NHE9-GFP localization with Rab5 in U87 cells was done using Manders’ coefficient (0.51 ± 0.05. *n* = 50 cells). **b** Calibration of endosomal pH in U87 cells (**c**) pH in sorting endosomes is acidified in U87 cells transfected with miR-135a relative to scrambled control. Graph represents mean from three biological replicates and at least 50 cells were used for pH quantification in each experiment. Error bars represent standard deviation (SD); *p < 0.05. Statistical analysis was done using student’s t-test

To determine the mechanistic basis of miR-135a directed regulation, we confirmed the subcellular localization of NHE9 in U87 cells. NHE9-GFP colocalized with Rab5, a marker for sorting endosomes (Manders’ coefficient, 0.51 ± 0.05 S.D., *n* = 50 cells) (Fig. 4A). Previously, NHE9 has been shown to leak protons out of the endosomes in glial cells. Therefore, we expected a decrease in NHE9 protein expression by miR-135a to result in more acidic endosomes. To this end, we measured luminal pH of the sorting endosomes (pH_e_) using pH-sensitive fluorescence of FITC-tagged transferrin. There was no significant effect of miR-135a transfection on recruitment of transferrin to the sorting endosomes (Additional file 1: Figure S2). pH_e_ was calibrated using buffers of known pH (Fig. 4B). In U87 cells, as expected pH_e_ changed from 5.95 ± 0.12, to 5.63 ± 0.14 upon transfection with mir-135a mimic (Fig. 4C).

**Fig. 5:**
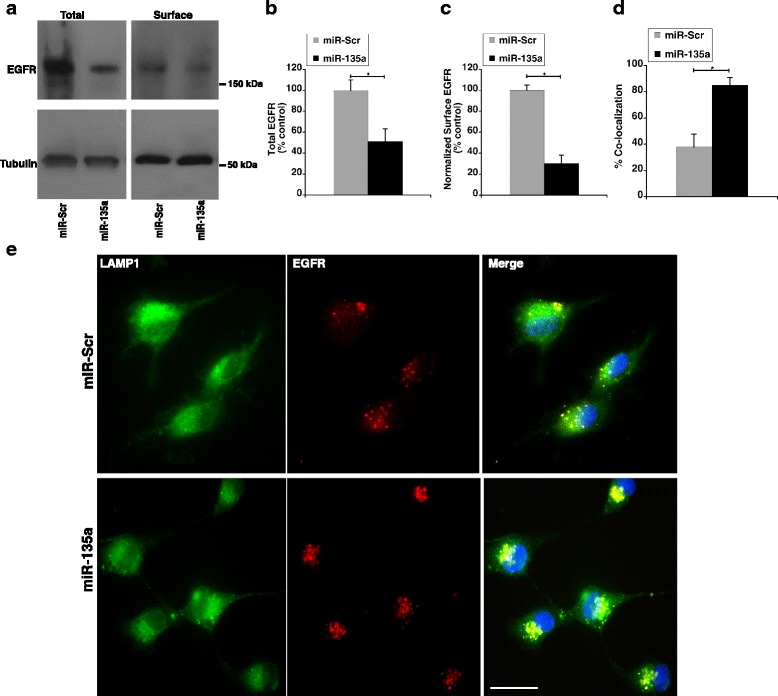
miR-135a regulates NHE9 to limit number of EGF receptors on cell surface. **a** Immunoblot showing total and plasma membrane epidermal growth factor receptor (EGFR) expression levels from U87 cells transfected with miR-135a or scrambled control. EGFR expression on plasma membranes was determined by surface biotinylation (**b** and **c**) Graphs represents average band intensity from densitometric scans of immunoblots from three biological replicates. Surface EGFR levels were normalized to total EGFR protein. MiR-135a not only downregulates EGFR expression but also limits EGFR presence on the cell surface by inhibiting NHE9 expression. **d** Quantification of colocalization between LAMP1 and EGFR signals is shown in the graph (*n* = 30 cells). Signal intensity and colocalization were measured with Image J software. Error bars represent standard deviation (SD); *p < 0.05. Statistical analysis was done using student’s t-test. **e** Colocalization of LAMP1 (green) and EGFR (red) images from immunofluorescence staining in U87 cells transfected with miR-Scr and miR-135a. DAPI staining is shown in blue. Colocalization is indicated by yellow in the merge. Scale bar is 50 μm

**Fig. 6:**
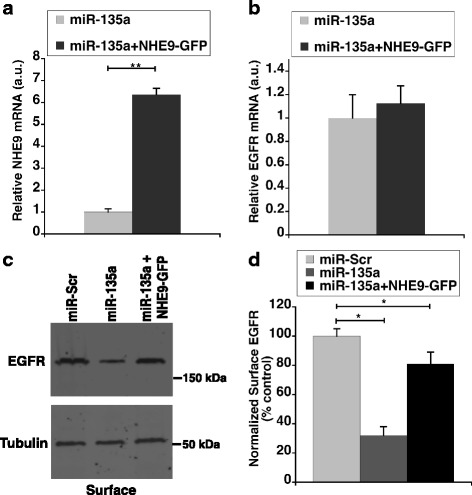
Ectopic NHE9 expression in miR-135a transfected cells rescues plasma membrane EGFR expression. **a** Relative increase in NHE9 transcript upon ectopic expression of NHE9-GFP in miR-135a transfected U87 cells determined by quantitative real-time PCR analysis (**b**) No significant change in EGFR transcript is observed in miR-135a transfected U87 cells upon ectopic expression of NHE9-GFP (**c**) Immunoblot showing rescue of epidermal growth factor receptors (EGFRs) on cell surface upon ectopic NHE9-GFP expression in U87 cells transfected with miR-135a. EGFR expression on plasma membranes was determined by surface biotinylation (**d**) Graph represents average band intensity from densitometric scans of immunoblots. Surface EGFR levels were normalized to total EGFR protein. All graphs represent an average of at least three biological replicates. Error bars represent standard deviation (SD), *p < 0.05 and **p < 0.01. Statistical analysis was done using student’s t-test

pH in sorting endosomes is crucial for receptor sorting and turnover. EGF receptor mediated signaling is a powerful driver of glioblastoma. EGF binding to the receptors on the cell surface activates downstream kinase cascades responsible for uncontrolled cell proliferation. However, drugs designed to inhibit receptor kinase phosphorylation have not been very successful due to redundancy in signaling pathways and constitutively active mutations. An alternative strategy to explore is decreasing EGFR availability on the cell surface by manipulating receptor turnover by altering the luminal pH of sorting endosomes. We therefore, sought to determine the effect of NHE9 downregulation via miR-135a transfection on plasma membrane localization of EGFRs in U87 cells. To this end, we first examined the effect of miR-135a on total cellular EGFR expression. Western blot analysis indicated cellular EGFR expression decreased by ~50% in miR-135a transfected U87 cells relative to control (Figs. 5A and B). This is consistent with a previous study in prostate cancer cells, which showed miR-135a directly targets EFGR transcripts to downregulate their expression [38]. Furthermore, it was previously shown that elevated expression of NHE9 limits EGFR degradation [7]. Therefore, the total decrease in EGFR protein we observed could be a combination of transcript downregulation by miR-135a and increased protein degradation. Next, in EGF stimulated U87 cells we used surface biotinylation to determine the plasma membrane density of EGFRs. Compared to control, we observed ~70% decrease in EGFR surface expression in miR-135a transfected U87 cells, after normalizing for total cellular EGFR expression (Figs. 5A and C). In addition to downregulating EGFR expression in glioblastoma cells, our data suggest that miR-135a affects EGFR turnover. To confirm this, we used immunofluorescence microscopy to examine localization of activated EGFRs with lysosomal marker LAMP1 in miR-135a transfected U87 cells. Consistent with miR-135a expression promoting sorting of EGFRs for lysosomal degradation, we observed a significant increase in colocalization of EGFR with LAMP1 in miR-135a transfected cells (Manders’ coefficient, 0.85 ± 0.06 S.D., *n* = 30 cells) relative to scrambled control transfected cells (Manders’ coefficient, 0.38 ± 0.10 S.D., n = 30 cells) (Figs. 5 D-E). To demonstrate that differences in EGFR turnover are linked to NHE9 levels, we ectopically expressed NHE9-GFP in U87 cells transfected with miR-135a following which we conducted experiments to quantify EGFR levels on cell surface. Ectopic expression increased NHE9 transcript levels by ~ 6.5 -fold (Fig. 6A). NHE9-GFP transduction had no significant effect on total EGFR expression (Fig. 6B ). Though there was no significant change in EGFR transcript levels in miR-135a transfected U87 cells overexpressing NHE9 (Additional file 1: Figure S3), greater than 50% of EGFR plasma membrane expression was rescued in these U87 cells (Figs. 6C and D).

Taken together, our data clearly show that reduced EGFR surface expression is causally related to the observed proliferative and migratory phenotypes upon miR-135a expression and NHE9 downregulation. Notably, NHE9 expression can suppress the effect of miR-135a on cell growth and migration to a large extent, suggesting that the effect of miR-135a mediated EGFR downregulation on these phenotypes could be limited.

## Discussion

NHE9 is implicated in multiple neurological diseases including glioblastoma. However, regulation of its expression has not been reported till date. Here, we show for the first time that miR-15a is a regulator of NHE9 expression. In glioblastoma, overexpression of NHE9 is a potent driver of tumor progression and is associated with decrease in patient survival [7]. Understanding molecular mechanisms altering NHE9 expression may lead to more effective therapeutic and diagnostic approaches. At a molecular level, mechanisms affecting transcription, translation, rate of protein degradation or combinations of these are potentially responsible for increase in NHE9 levels beyond a critical threshold. We propose attenuation of miR-135a mediated downregulation of NHE9 as a possible mechanism for NHE9 overexpression in glioblastoma. There is mounting evidence implicating miRNA alterations in cancer initiation and progression [18–20]. MicroRNA-135a is enriched in glia and is negatively correlated with the pathological grading in gliomas [28]. NHE9 is one of the highly expressed proteins in high-grade (grade IV) glioma [7]. Thus, it is possible that loss of post-transcriptional regulation via miR-135a could lead to NHE9 overexpression. Each gene may be regulated by more than one miRNA [24]. Consistent with this, we show that NHE9 is a putative target of both miR-124 and miR-135a. Among miRNAs profiled in malignant gliomas, miR-124 stands out to be one of the better-characterized miRNAs. MiR-124 is the most abundant miRNA in the brain and is expressed at significantly lower levels in glioblastoma compared with non-neoplastic brain tissue [39–41]. Ectopic expression of miR-124 in U87 cell line resulted in significant inhibition of migration and invasion [39]. However, role for miR-124 in regulation of NHE9 expression awaits further experimentation.

**Fig. 7:**
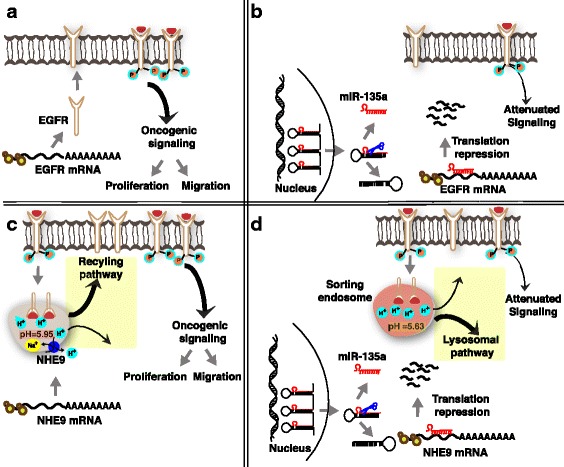
Model for two-pronged regulation of EGFR signaling by miR-135a. Downregulation of EGFR and NHE9 expression in U87 cells by miR-135a affects glioblastoma cell proliferation and migration. miR-135a reduces the total number of EGFRs by downregulating total cellular EGFR expression and limits the (already) low EGFRs from reaching plasma membrane by downregulating NHE9 expression. **a** In U87 glioblastoma cells, miR-135a expression is downregulated. EGFR transcript is translated in the cytosol and the receptors are transported to the cell surface. EGF binding activates downstream signaling, which could turn oncogenic due to EGFR persistence on the plasma membrane leading to increased cell proliferation and migration. **b** Upon expression of miR-135a gene, pre-miRNA 135a is transported out of the nucleus and is processed in the cytoplasm resulting in mature miR-135a. Mature miR-135a binds to the 3’UTR of EGFR transcript resulting in degradation of the mRNA. As a consequence of overall decrease in EGF receptors, oncogenic signaling is attenuated resulting in decreased cell proliferation and migration. **c** Endocytosed receptors are sorted in the early/sorting endosome either for recycling or degradation in the lysosome. Luminal pH in the sorting endosome regulates the route of their cargo. pH in these endosomes is governed by pump-leak mechanisms [7]. NHE9 expression allows for protons to leak out in exchange for Na^+^ or K^+^ ions, thereby alkalinizing the lumen (pH = ~5.95). EGFR is trafficked in pH-defined endosomal compartments within the cell. Increased leak due to NHE9 expression has been shown to recycle EGFR receptors back to the cell surface [7]. **d** In this model, we show that downregulation of NHE9 expression via miR-135a acidifies the pH in the lumen of sorting endosomes (pH = ~5.63). Loss of NHE9 activity diverts the EGF receptors away from the plasma membrane towards the lysosomes, thus decreasing the number of EGFRs available for oncogenic signaling

EGFR signaling pathways are promising targets for therapeutic intervention in glioma [42]. However, EGFR-targeting therapeutics have not been very effective. Redundancies in downstream signaling pathways, adaptive resistance of the receptor to inhibitors have been attributed to the failure of these interventions. Regulating NHE9 expression via miR-135a based therapeutics could be explored to circumvent problems associated with EGFR persistence on the membrane. Our data supports the idea of a two-pronged approach to regulation of EGFR signaling by miR-135a (Fig. 7A-B). We show that ectopic expression of miR-135a in U87 glioma cells downregulates EGFR expression. Furthermore, miR-135a expression modulates endosomal pH by targeting NHE9 in U87 cells. Targeting endosomal pH limits trafficking of the remaining few EGFR molecules to the plasma membrane enhancing the efficiency of regulation (Fig. 7C-D). As a consequence, up-regulation of miR-135a suppresses proliferation and migration of glioblastoma cells. Evidence for a similar multi-pronged approach to targeting proteins in a single pathway by miR-135a has been reported in the mitochondria-dependent apoptotic pathway where STAT6, SMAD5 and BMPR2 were shown to be direct targets of miR-135a [28, 38]. Silencing these three target genes was shown to contribute to greater caspase-3/7 activity in glioma.

Multiple miRNA-based therapeutics have reached clinical development. In the context of miRNA therapy, an important factor to consider is miR-135a’s functional heterogeneity in different cell types. For example, in melanoma cells mir-135a is upregulated resulting in downregulation of the forkhead box O 1(FoxO1) protein, a transcription factor known for its tumor suppressor role [43]. Here, miR-135a functions more like an onco-miRNA turning on oncogenic signaling pathways via FOXO1 repression. Therefore, systemic administration of miR-135a in the body may not be tolerated. However, there are no known reports of intolerance in other brain cell types to miR-135a.

## Conclusions

In conclusion, we identify for the first time a regulator for NHE9, implicated in multiple neurological diseases. We propose downregulation of miR-135a that is normally enriched in glial cells as a potential mechanism leading to increase in NHE9 expression beyond a critical threshold, which drives oncogenic signaling in a subset of glioblastomas. Taken together, our findings indicate miR-135a is compelling target for therapeutic development in NHE9 overexpressing glioblastoma.

## Additional file

Additional file 1: Supplementary Material. (DOCX 1304 kb)

## Abbreviations

EGFR: Epidermal Growth Factor Receptor
GBM: Glioblastoma multiformae
MIR: MicroRNA
NHE9: Na^+^(K^+^)/H^+^ Exchanger isoform 9
