# Supplementary material for: MicroRNA-135a regulates NHE9 to inhibit proliferation and migration of glioblastoma cells
Source: Cell Commun Signal. 2017 Dec 21;15:55. doi: 10.1186/s12964-017-0209-7 (PMC5740897; doi:10.1186/s12964-017-0209-7)
Supplement: Supplementary file 1 — Supplementary Material. (DOCX 1304 kb) [file 12964_2017_209_MOESM1_ESM.docx]

**Supplementary Material**

**Supplementary Figure 1**


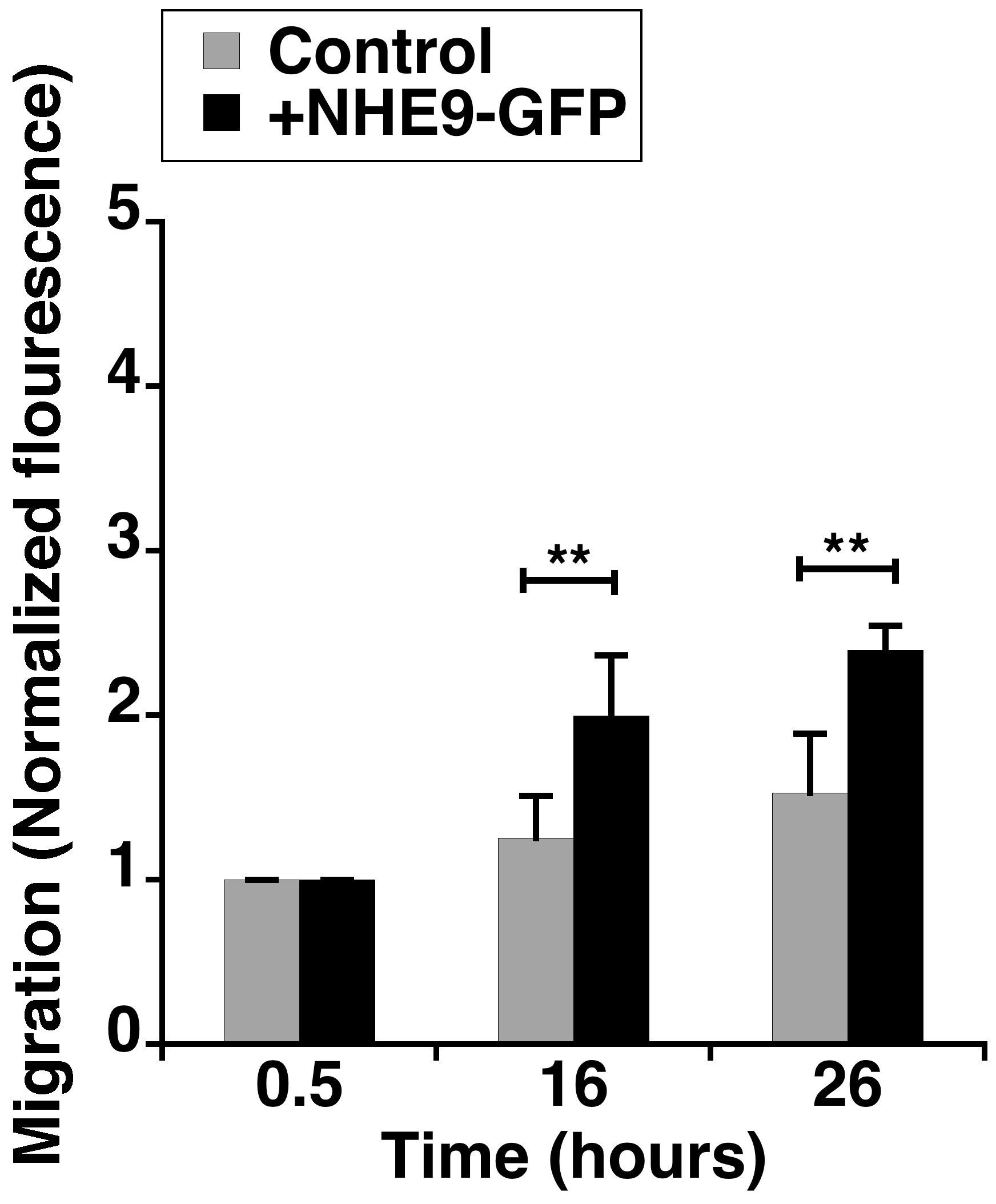


Graph represents normalized fluorescence intensity from NucBlue staining of live U87 cells migrating into the cell free zones at various time points as indicated. Fluorescence intensity of each sample was normalized to the samples fluorescence at 0.5 hours. Error bars represent standard Deviation (SD); **p<0.01. Statistical analysis was done using student’s t-test. Graph represents an average of at least three biological replicates.

**Supplementary figure 2**


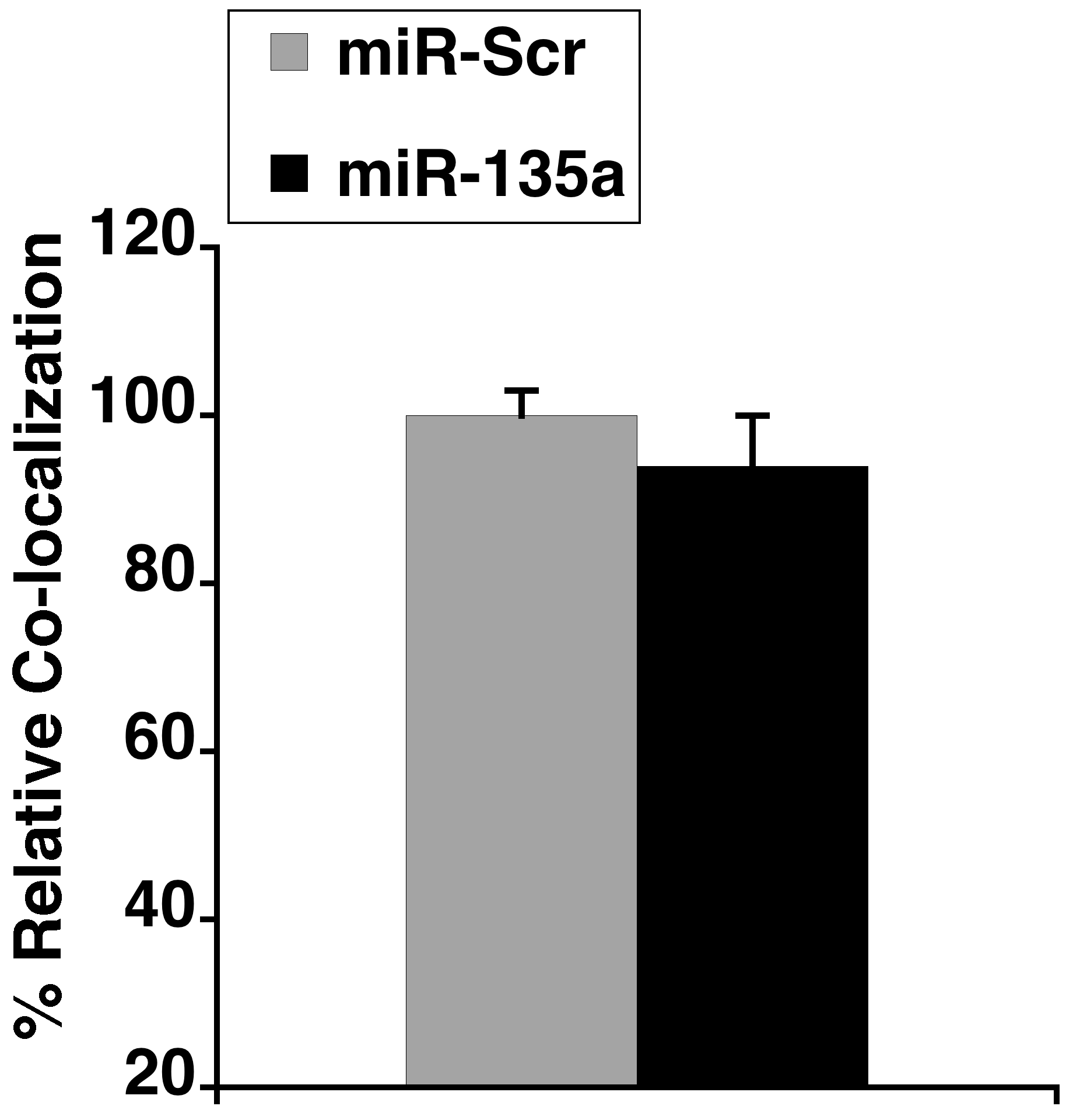


Relative colocalization (%) of transferrin in control and miR-135a transfected U87 cells, based on quantification of transferrin and Rab5 colocalization using Manders coefficient. Error bars represent standard deviation (SD); Statistical analysis was done using student’s t-test.

**Supplementary Figure 3**


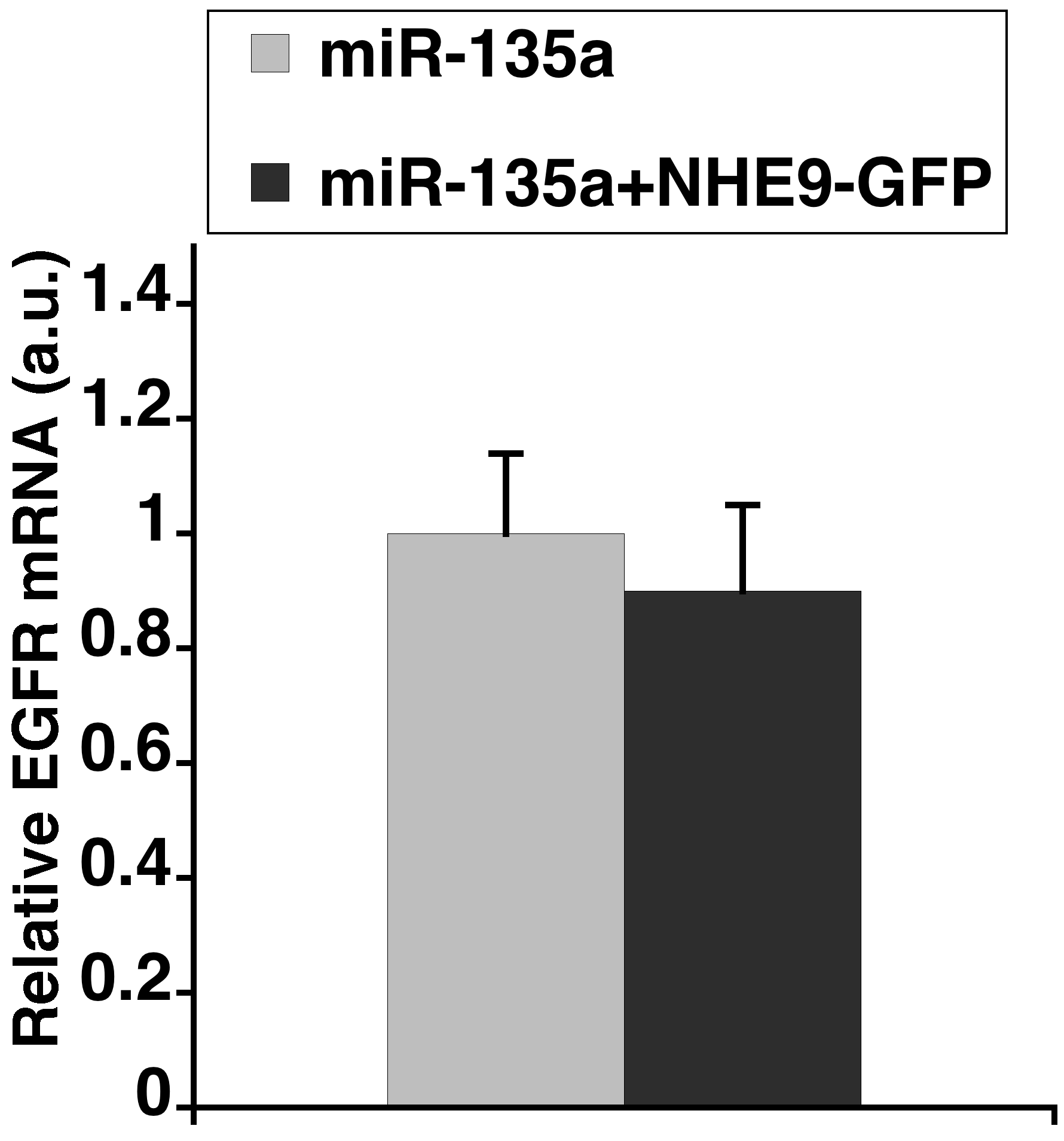


qPCR analysis of EGFR transcript levels in miR-135a transfected cells overexpressing NHE9 relative to control cells. Graph represents an average of three biological replicates.
